# Intestinal Parasites, Anemia and Nutritional Status in Young Children from Transitioning Western Amazon

**DOI:** 10.3390/ijerph17020577

**Published:** 2020-01-16

**Authors:** Rejane C. Marques, José V. E. Bernardi, Caetano C. Dorea, José G. Dórea

**Affiliations:** 1 Instituto de Biofísica Carlos Chagas Filho Universidade Federal do Rio de Janeiro, Rio de Janeiro 21941-901, Brazil; rejanecmarques@globo.com; 2 Department of Nutrition, Universidade de Brasília, Brasilia 70910-900, Brazil; bernardi.jve@gmail.com (J.V.E.B.); jg.dorea@gmail.com (J.G.D.); 3 Department of Civil Engineering, University of Victoria, Victoria, BC V8P 5C2, Canada; 4 Environment & Regional Development Graduate Program (PGDRA), Universidade Federal de Rondônia (UNIR), Porto Velho 76801-974, Brazil

**Keywords:** Amazon, anemia, fish consumption, hair mercury, hemoglobin, parasites, sanitation

## Abstract

Young children are particularly vulnerable to the chronic sequelae of anemia, including poor nutritional status. The aim of this study was to assess intestinal parasitic-infections and nutritional status (anemia and linear growth) in preschool children living in contemporary Amazonian communities. A cross-sectional study measured children’s intestinal parasites and hair-Hg (HHg)—biomarkers of fish consumption, hemoglobin levels, and growth (anthropometric Z-scores). Children came from traditional-living families (Itapuã), and tin-mining settlements (Bom Futuro) representing current transitioning populations. It covered 937 pre-school children (from 1 to 59 months of age) from traditional (247) and immigrant tin-mining families (688). There was a high prevalence of intestinal polyparasitic-infection in children from both communities, but mild anemia (hemoglobin concentrations) and moderate (chronic) malnutrition were more frequent in children from traditional families than in children from tin-mining settlers. Children from traditional families ate significantly more fish (HHg mean of 4.3 µg/g) than children from tin-mining families (HHg mean of 2.3 µg/g). Among traditional villagers, children showed a significant correlation (r = 0.2318; *p* = 0.0005) between hemoglobin concentrations and HHg concentrations. High rates of parasitic infection underlie the poverty and attendant health issues of young children in the Brazilian Amazon. The intestinal parasite burden affecting poor Amazonian children resulting from unsafe water, lack of sanitation and poor hygiene is the most urgent environmental health issue.

## 1. Introduction

The World Health Organization (WHO) estimates that anemia affects two billion people all over the world. Due to their increased nutritional requirements for iron (Fe), young children are particularly vulnerable to the chronic sequelae of anemia (long-term biological consequences and social impacts)—reduced immunity (and attendant morbidity) and cognitive delays that can compromise intellectual development [1]. Amazonian rivers are an abundant source of fish for traditional communities, and fish provide a large proportion of the protein and essential nutrients consumed by subsistence villagers in the Amazon rainforest [2].

The etiology of anemia in preschool children of poor socioeconomic background is multifactorial and complex: it includes the dietary insufficiency of micronutrients such as Fe and vitamins (A, B12, folate and riboflavin). Indeed, among environmental factors, diet has been strongly associated with anemia. Cowin et al. [3] reported that the prevalence of low hemoglobin (Hb) levels was higher in 18-month-old children who consumed no animal protein. Rodrigues et al. [4] reported that Hb was significantly associated with bioavailable dietary Fe in children aged 6 to 24 months. Furthermore, young children (15 to 18 months) with Fe deficiency showed higher intake of cow’s milk and lower intake of animal protein that included fish [5]. Indeed, fish consumption has been positively associated with ferritin stores in children [6,7].

It is known that fish protein enhances non-heme Fe uptake from diets [8]. Studies have consistently shown that dietary fish enhances the absorption of Fe from plant foods [9,10]. The addition of 40 g of fish to a rice-and-vegetable-based diet of Southeast Asian adults increased non-heme-Fe absorption by 6% and 11.9%, respectively [11]. Recent studies have shown enhanced Fe-bioavailability as a result of fish-increased Fe absorption [12,13]. Additionally, cassava, the staple food of Amazonians, has been reported as an important source of dietary Fe [14].

With the expansion of agriculture and mining activities, Western Amazonia has experienced rapid growth. This, in turn, has attracted new inhabitants and has altered traditional riverine living. One of the lifestyle changes is in the diet, with fish being replaced by other protein sources, thus also modifying attendant nutrients related to a fish-based diet. Studies addressing the nutritional status of children in the Amazon region have shown that replacing fish protein has not impacted linear growth within communities [15,16,17,18,19]. However, given the role of dietary fish on Fe metabolism, it is not yet established how the prevalence of children’s anemia has been influenced by changes in fish consumption. 

Besides nutritional deficits due to food insecurity, parasitic infections attendant on substandard living conditions can act individually or in combination to aggravate anemia. Traditional living in riverine populations has been characterized by a high incidence of intermittent malaria (with hemolytic crisis) and endemic lifelong infestation with intestinal parasites [20]. Studies in Asia, Africa, and Latin America reviewed by Guerrant et al. [21] have shown that entero-aggregative *Escherichia coli*, *Cryptosporidium* and *Giardia* are among the leading pathogens that cause persistent diarrheal illnesses; additionally, they summarized data showing also that intestinal helminthic infections impair intestinal function (nutrient absorption), with negative effects on growth and nutritional status. Generally, in Amazonian settlements there are inadequate sanitation facilities to safely dispose of excreta, resulting in the contamination of the surrounding environment and risk of excreta-related diseases [22].

The objective of this work was to assess intestinal parasite infestation and hemoglobin levels, and to compare nutritional status in under-five children representing populations with differences in social and fish-eating habits (traditional riverine communities and tin-mining migrants).

## 2. Materials and Methods 

### 2.1. Background Information

This study is part of a larger research program designed to evaluate children’s health and nutritional status due to economic and social changes occurring as a result of occupation of the Western Amazon. The study protocol was approved by the Ethics Committee for Studies in Humans of the “Universidade Federal de Rondonia” (Of. 001-07/CEP/NUSAU). Research followed ethical recommendations guaranteeing written consent (with voluntary participation and assured confidentiality) by the participant mother, who could withdraw from the study at any time. The study was explained to the mother and an informed consent form was obtained and signed by all the volunteering mothers. The recruitment of mothers in the rural villages has been described in Marques et al. [17] and Marques et al. [18]—there were no exclusions or dropouts. All infants younger than five years of age were enrolled for this study. Socio-demographic data from the family and pertinent information on the infant were collected by interviewing each mother. To ensure standardization during interview, all questions were read aloud to the mother.

**Figure 1:**
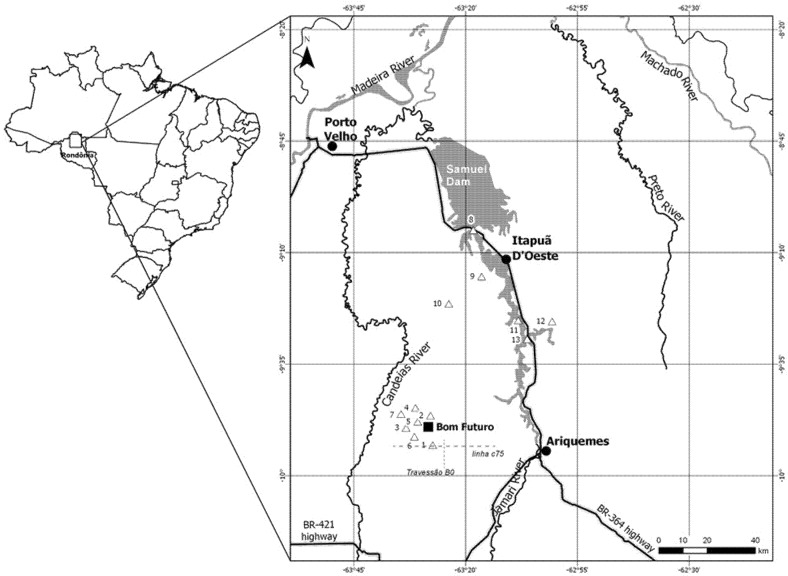
Map of the region showing points of sample collection for the rural localities of Bom Futuro and Itapuã; adapted from Marques et al. [17,18].

Training of the research team occurred prior to the survey and supervision was exerted to maintain standardization of the data collection procedures at all times. All children in the designated localities were surveyed (Figure 1), with complete information on anthropometry and used total hair-Hg (HHg) concentrations as a marker of fish consumption. The survey was carried out through several visits between February 2006 and December 2007. During visits, trained professionals conducted interviews and collected samples of hair, stools, and performed a finger-prick blood-test to obtain hemoglobin (Hb) values. The hemoglobin assessment was done with a portable HemoCue^®^ photometer (hemoglobinometer, Angelhoim, Sweden) after calibration according to the manufacturer’s instructions. The results were expressed in grams per deciliter (g/dL). Children were considered anemic if their hemoglobin levels were below 11 g/dL [23]. The detailed protocol and methods for anthropometry and HHg determinations have been described in a parent publication [17,18]. Briefly, a questionnaire was administered to the mothers by trained interviewers in order to acquire demographic data; questions pertained to the household environment and social variables as well as the frequency of fish consumption. The birth certificate was used to obtain the children’s age.

### 2.2. Hair Sampling and Total Hair-Hg Determination

Total hair-Hg concentration was used to complement fish intake information. A hair sample was cut close to the scalp (occipital area) from the children using a stainless-steel scissors. The hair was placed in an envelope, properly identified, and taken for analysis in the Radioisotopes Laboratory of the Federal University of Rio de Janeiro.

All glassware used was washed clean and rinsed with solutions of 5% (*w*/*v*) ethylenediaminetetraacetic acid (EDTA) and double distilled water; the clean material was left to rest in 5% (*w*/*v*) HNO_3_ overnight. Then a final rinse in double-distilled water and it was left to dry at 100 °C for 12 h. The preparation and analytical procedures of the hair samples followed the standardized laboratory procedures. The hair samples were cut into small pieces, washed with EDTA at 1% (*w*/*v*) and properly dried at 50 °C; after that, samples were digested at 80 °C for 40 min with concentrated HNO_3_ (3 mL) and KMnO_4_ (6 mL, 5% (*w*/*v*)) in a microwave oven (MDS 2000; CEM Corporation, Matthews, NC, USA) for 35 min. The total Hg concentrations were determined by cold vapor atomic absorption spectrometry with a flow injection system (CV-AAS, FIMS 400; Perkin-Elmer, Ueberlingen, Germany). Precision and accuracy were assessed with Hg internal standards, triplicate analyses, and certified reference materials (IAEA-085 and 086, Vienna, Austria) with recoveries of 92% [17,18].

### 2.3. Nutritional Evaluation: Anthropometry and Hemoglobin Measures

Weight and height (length in young children) were measured by trained personnel according to standard procedures described elsewhere [17,18]. Briefly, youngsters dressed in light clothing and barefoot were measured and weighed to the nearest 0.1 kg and to the nearest 0.1 cm, respectively. Weight (kg) and height (cm) were used to calculate Z-scores for attained weight-for-age (W/A), height-for-age (H/A), and weight-for-height (W/H) employing the WHO-Anthro (version 2; Department of Nutrition, World Health Organization, Geneva) and the WHO recommended growth curves [24]. In Marques et al [17], the children from Itapuã were studied with regard to traditional living (marked by habitual high fish consumption and attendant methylmercury exposure) and its impact on neurodevelopment. In Marques et al [18], children from families living on a tin-mining area and having a non-traditional life-style, likely were exposed to pollution occurring in mining environments and also consuming fish that is abundant in the region; in this study, the main objectives were pre-school neurodevelopment. We took advantage of these studies to address differences in children growth and family characteristics; for this end, we combined data from the two studies and constructed Table 1 in order to statistically analyze differences between the villages.

Hemoglobin concentration was measured in the field from a finger-prick blood sample with a portable ß-hemoglobinometer (HemoCue, Angelholm, Sweden) following standard procedures. Not all children could provide Hb measurements; among Itapuã villagers 221 were obtained (out of 247) and 535 (out of 688) in Bom Futuro. The anemia diagnosis followed international guidelines for severe (<7 g/dL), mild (7.0 to 9.9 g/dL), and moderate (10 to 11 g/dL) for the age category of 0 to 59 months [25].

### 2.4. Intestinal Parasites

During visits, families were given plastic vessels for fecal sample collection, along with instructions and recommendations on how to handle and store the fecal material safely. Vessels containing recommended preservative solution of mercury-iodine-formol were previously identified for each child. Three samples collected on different days were put in the same vessel and sent to Rondonia State laboratory for analysis. Standard procedures [26] to detect intestinal parasites were those of Hoffman-Pons-Janer [27]. The results of the tests were immediately conveyed to the families, and positive cases were instructed to seek the nearest health service for treatment. All families received educational materials and counseling regarding basic and community hygiene related to water treatment, fecal waste disposal, and garbage disposal.

To complement environmental information regarding parasitic infection, the presence of sanitation facilities for domestic fecal disposal was observed. Besides open defecation (a very common practice among young children), family disposal of feces is mainly done in latrines. These rudimentary facilities (“casinhas”) are generally separated from the main building and consist of a wooden cubicle built over a dug-out pit to contain human wastes.

### 2.5. Statistical Analysis

Data were summarized (means, standard deviation) and correlation analysis was conducted using Prism software (Prism, version 10IC; GraphPad Software Inc., San Diego, CA, USA); this statistical package was used to generate graphs and run Spearman’s (*p*) correlation between the variables of interest. The Kolmogorov-Smirnov one-sample was applied to test for normality of data and the Mann–Whitney U Test was used to test differences between villages (Statistica statistical package v.7.0; StatSoft, Inc., Tulsa, OK, USA).

## 3. Results

Table 1 summarizes the general characteristics of the subjects. Except for some shared characteristics related to family income, maternal education, and intestinal parasitic infestation, most of the variables related to children were significantly different between these populations. The fishing families of Itapuã tended to maintain some of the traditions related to extensive breastfeeding and habituation of fish consumption. They tended to breastfeed for longer periods and their children showed a higher mean HHg.

Children from Itapuã were older and, as a result, showed a higher mean breastfeeding duration (11.9 months). These children also showed a mean HHg concentration (4.3 µg/g) that was significantly higher than in children from Bom Futuro (2.4 µg/g). While differences in HHg concentrations were due to frequency of fish consumption, the higher mean duration of breastfeeding could be the result of sampling bias; Itapuã children were older than Bom Futuro children. Indeed, means of both height and weight were also higher for the Itapuã children. The complete study of the anthropometric status of these two groups of children appeared in two independent publications by Marques et al. [17,18]. However, differences in birth weight favoring children of tin-miner families showed a slightly better nutritional status (Z-scores for W/A and H/A). Moderate stunting was more prevalent in the Itapuã children; however, moderate under-weight was similar for both communities. In both groups, most of the children showed HHg concentrations above 1 µg/g (100% for Itapuã and 99% for Bom Futuro); HHg levels above 5 µg/g were higher among the children of Itapuã (25%) than in children from Bom Futuro (1.4%).

**Figure 2:**
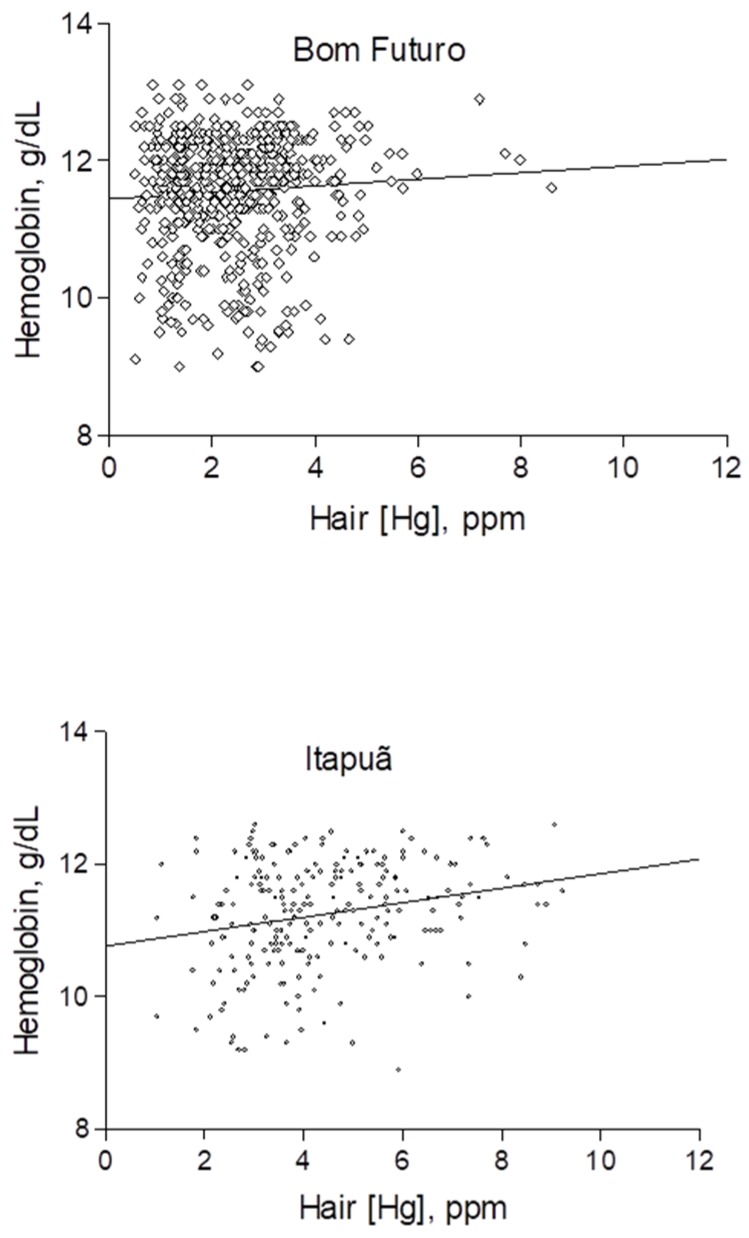
Correlation between hair-Hg concentrations (a marker of fish consumption) and hemoglobin (Hb) concentrations according to villages.

The children from Bom Futuro (tin-miners) also showed a significantly higher mean Hb concentration (Table 1). The frequency of mild and moderate anemia (Hb < 11.0 g/dL) was higher for children from Itapuã (33%) than for children from Bom Futuro (20%). Except for one child from Itapuã, there were no cases of severe anemia (Hb < 7.0 g/dL). The children from Bom Futuro showed a slight but significantly higher Hb concentration (11.6 g/dL) than the children from Itapuã (11.2 g/dL). However, correlation between fish consumption (HHg) and Hb concentration (Figure 2) was statistically significant for children from Itapuã (r = 0.2318; *p* = 0.0005) but not for children from Bom Futuro (r = 0.0663; *p* = 0.1258).

**Figure 3:**
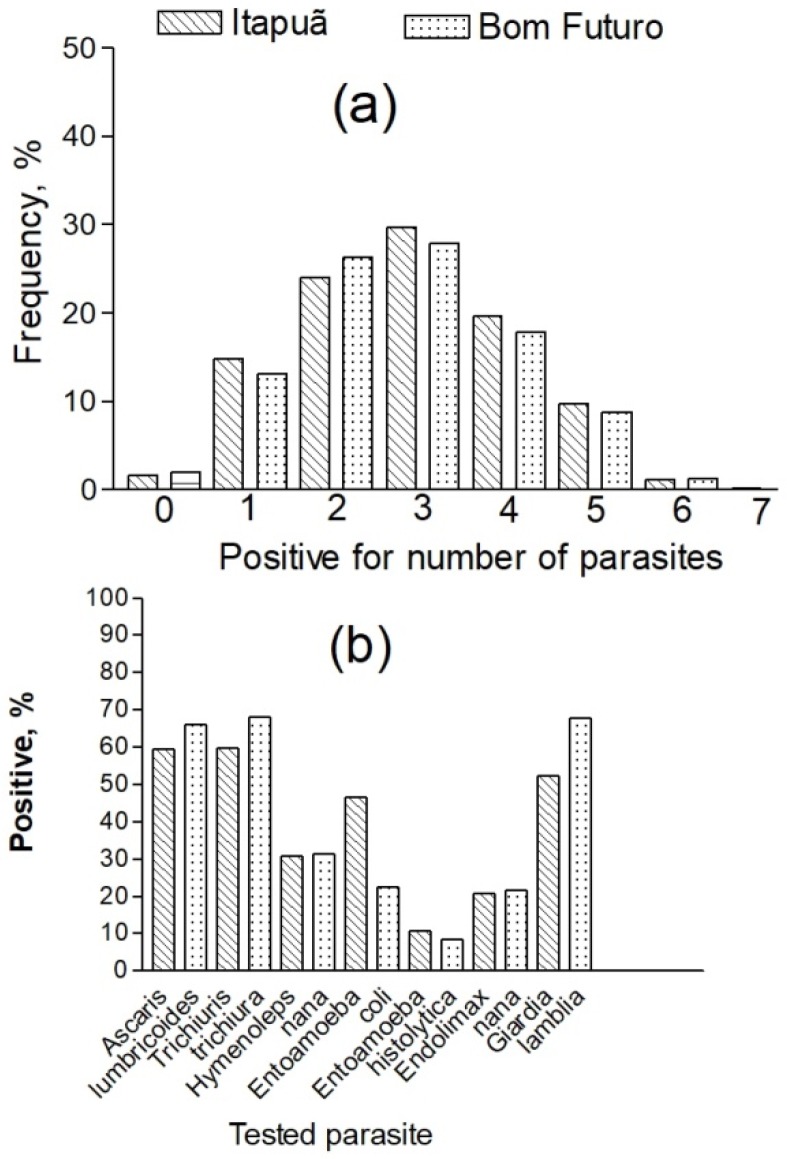
Illustration comparing fishermen (Itapuã) and tin miners (Bom Futuro) as a function of (**a**) frequency of children testing positive for a type of parasite; (**b**) frequency of intestinal parasites found in children.

As shown in Figure 3, a total of seven different intestinal parasites were found: three worms (*Ascaris lumbricoids*, *Trichuris trichiura*, *Hymenolepis nana*) were diagnosed based on the detection of eggs in feces, and four protozoans (*Giardia lamblia*, *Entamoeba coli*, *Entamoeba histolytica*, *Endolimax nana*). These intestinal parasites were found in both groups, and the mean prevalence was similar for both groups in most cases. Children testing negative for all parasites were a small proportion (<2%) in both groups; one or more (up to seven) parasites tested positive in 98% of children. Overall, most children (circa 60%) had at least 3 types of intestinal parasites; the rate of polyparasitic infection (>2) was slightly higher for the children of Itapuã. However, there was no significant difference in the number of positive tests for intestinal parasites between the two groups (Table 1).

The prevalence of testing positive for helminthes was very high (Figure 3), but the most prevalent intestinal parasites were *A. lumbricoides*, *T. trichiura*, and *G. lamblia* (ranging from 52.2% to 68.2%). Except for *Ent. coli* (Itapuã: 46.6%; Bom Futuro: 22.5%) the overall prevalence was similar: helminthes (*A. lumbricoides*, *T. trichiura*, *H. nana*) and protozoan (*G. lamblia*, *Ent. histolytica*, *E. nana*) infections were found in similar proportions in both communities. However, children from Bom Futuro showed a slightly higher prevalence for helminthes. Most children (68.2%) harbored a helminthes species (*T. trichiura*).

## 4. Discussion

The high prevalence of intestinal parasites is an important finding, drawing attention to the poor environmental conditions and neglect that threaten the future of these Amazonian children. In the social transitioning that is taking place in Western Amazonia, moderate anemia (Hb < 11.0 g/dL) is high and unequally distributed between the two communities. Both moderate anemia and mild undernutrition showed a higher frequency in children from Itapuã than in children from Bom Futuro. This indicates that there are environmental modifying factors differentiating these groups. Among the measured differences in characteristics of these communities are higher family fish consumption and lower birth weight observed in the Itapuã group. Additionally, it compares two different populations and lifestyle (traditional living and tin-mining immigrants). The comparison revealed statistically significant differences in family structure and lifestyle (fish consumption, family size, and breastfeeding practices) as well as in children measured parameters (birth length, age at visit, weight, height, W/H Z-score, H/A Z-score, Hb, and hair-Hg).

The co-existence of poor sanitation and unclean water is a common finding among poor Amazonians; indeed, the lack of hygienic facilities to separate fecal material from ground water contamination is a problem that affects the poor around the world [28]. Although high prevalence for both *Ent. coli* and *Ent. histolytica* was found among the identified intestinal parasites, it should be noted that the former is not considered pathogenic, but it is indicative of a poor hygienic environment. Indeed, the prevalence of *Ent. histolytica* was higher among the Itapuã children, where *Ent. coli* prevalence was almost twice that of Bom Futuro. *Ent. coli* is considerably more resistant than Ent. Histolytica [28], leading to its higher prevalence. Intestinal-worm infection (*A. lumbricoides* and *T. trichiura*) rates were similar for both populations. However, the overall rates in this study were higher than those reported for the city of Manaus [29]. Indeed, the prevalence of *A. lumbricoides* (>60%) is three times the threshold indicated by the World Health Organization [1]; therefore, this level of helminthic infections calls for aggressive control efforts. It was also noted that the parasites identified in this study shared the same fecal-oral transmission route [22]. Another study in Amazonian communities [29] revealed a comparable prevalence of *A. lumbricoides* and *T. trichiura*, but a striking difference with regard to prevalence of *Necator americanus* (not detected in the present study), averaging 55% in children under five. Notably, infection from this excreted parasite is cutaneous [22], but it would be premature to speculate that the absence of *N. americanus* from stool samples in this study could be related to the high levels of sanitation coverage observed (Itapuã: 98%; Bom Futuro: 92%).

Although diarrheas were not accounted for, it is known that enteric infections (without overt diarrhea) can predispose children to nutritional shortfalls. In Mexican schoolchildren, Quihui-Cota et al. [30] reported a significant association between trichuriasis and hemoglobin; indeed, hemoglobin concentration rose significantly in the infected children 5 weeks after anti-helminthic treatment (*p* < 0.05). Compared to the present study, Ecuadorian school-aged children of the Amazon have showed a relatively lower (16.6%) rate of anemia [31]. 

Mean differences between anthropometric Z-sores were statistically significant for W/H and H/A, showing distinct profiles regarding mild undernutrition. Changes in fish consumption and lifestyle differences of current Amazonian populations showed that families’ shift from a fish-rich diet had no negative impact on the growth of these children [17,18]. Indeed, in the group with a wider difference in family fish consumption, the correlation between HHg (a marker of fish consumption) and anthropometric Z-scores was not statistically significant [18]. Concurring with our results, Maia et al. [29] also reported lower rates of undernutrition in Amazonian children that were not correlated with helminthes infection. In Colombian schoolchildren, Boeke et al. [32] reported that an asymptomatic protozoan (*G. lamblia*) was associated with H/A Z-score; they reported a prevalence of *Giardia* (6.3%) ten times lower than that in the present study (66%).

Although the role of food in anemia and undernutrition is not disputed, it is expected that intestinal parasites will compromise nutrient intake and absorption, and also act through persistent bouts of diarrheal diseases and enteropathies [21]. Indeed, stunting has been found to be associated with *Giardia* infection in Colombia [33], among the Israeli Bedouin [34], in Iran [35], in Ecuador [36], and in Turkey [37]. Quihui-Cota et al. [38] reported a significant interaction between helminthes infection (*H. nana* and *T. trichiura*) and the nutritional status of Mexican schoolchildren. Because most of the children in the present study had at least one parasite, only a small proportion of children had none (<2%) or tested positive for only one type of parasite (15%). It seems that among the children of Itapuã, the consumption of fish was beneficial to the Hb status; there was a positive and significant correlation between hemoglobin status and fish consumption (HHg). The use of hair-Hg as an indicator of fish consumption has been used before in Amazonian populations [2]. Subsistence communities—communities like the ones in the Rio Madeira Basin—are among the highest consumers of fish in the world; depending on the season and availability of fish, they consume a wide range of species with varied concentrations of Hg [2].

Given the opportunity to study children’s intestinal parasites in an environment of high fish consumption (and Hg exposure), it is worth speculating on the possible interactions of intestinal parasites and enteric methylmercury derived from fish intake. In early medical literature, mercury compounds were widely used to treat intestinal parasites [39]. Indeed, mercury biniodide was successfully used to treat balantidial dysentery [40]. Mercury has an affinity for intestinal-parasite eggs [41] and is used as a fixative in light microscopy. It is known from experimental studies that nematodes respond to mercury toxicity [42] and are also sensitive in accumulating mercury from host organisms [43]. The interplay of high rates of intestinal-parasite infections with high rates of intestinal methyl-Hg (derived from fish consumption) is one that has not yet been addressed in human ecology.

It is known from experimental studies that nematodes (*Caenorhabditis elegans*) are used as a model to study the mechanism of Hg toxicity [42], and this has also been shown to correspond to bioaccumulation of Hg in the aquatic food chain [43]. Bergey et al. [44] observed that non-parasitized fish tended to accumulate more Hg than their nematode parasites. Indeed, the synergy of protozoan parasites and methylmercury has been demonstrated in immunotoxicity and neurotoxicity studies in mice [45]. Therefore, susceptibility to diarrhea arising from enteric parasites needs to be studied in relation to high levels of fish-methylmercury in high fish-eating populations. The metabolic processes of gut microbiota could be affected by Hg enteric concentrations and enteric disease could be impacted by nutritional factors in fish.

This study will also help nursing and medical students to become aware of the magnitude of children’s vulnerability to environmental hazards. Its strength is that selection bias was avoided by enrolling all the children in the two communities, which are both representative of different lifestyles in the Amazon: (i) a former subsistence community that still relies on fish as a staple (with many households still depending on fishing), and (ii) tin-mining workers (new settlers). Although the data allowed comparison of the potential role of dietary fish, the study also tested for various intestinal parasites that included helminthes (which have been related to malnutrition and anemia). There are, however, several limitations. It was not possible to measure the individual parasite load (intensity of infection) in these children. Because only the presence of the parasite was measured, the severity of parasitic infection was not evaluated; thus, precluding the association of intestinal parasites and fish-methylmercury exposures. Furthermore, because stool samples were collected within short intervals, it was not established if the infection was recent or chronic. Another limitation is that only hemoglobin was measured, and the interpretation of low hemoglobin in children aged <6 months is recognized as difficult; additionally, other biomarkers of Fe status (such as ferritin) or micronutrients were not measured, and these could contribute to anemia (such as folate and vitamin B12) in children. 

## 5. Conclusions

High rates of parasitic infection underlie the poverty and attendant health issues of young children in the Brazilian Amazon. Compared to children from families living in tin-mining communities, linear growth and anemia status of children belonging to former riverine families are slightly more compromised. Intestinal parasite-infection rates—which result from unsafe water, lack of sanitation and poor hygiene—constitute a permanent threat to children’s health and are the most urgent environmental health issues. They are potentially more debilitating than the often-cited high exposure to fish-Hg experienced by poor Amazonian children. There are practical implications to be drawn from this study concerning environmental factors related to anemia and parasitic diseases: (a) such precarious sanitary conditions are not inevitable; (b) a clean environment with adequate sanitary disposal of feces and a clean water supply should be a sine qua non to control parasitic infection and avoid recontamination. Immediate societal and clinical treatment interventions to counteract co-morbidities associated with polyparasitic infection should be a priority for environmental health programs in the Amazon region.

## Figures and Tables

**Table 1:** General characteristics of families and children (937) comparing villagers of fishing community (Itapuã) with tin miners (Bom Futuro).

Communities	Itapuã Mean (SD)	Bom Futuro Mean (SD)	*p*-Level
Family			
Fish meal (week)	2.9 (2.3)	2.3 (1.4)	0.0000
Family members	5.8 (2.2)	5.11 (1.87)	0.0005
Family income (US dollar)	240.5 (211.2)	214.8 (134.2)	0.8225
Breastfeeding (months)	11.9 (8.9)	8.2 (7.7)	0.0000
Maternal education (years)	6.5 (3.5)	5.8 (2.7)	0.0714
Children (*n*)	249	688	
Birth			
Weight (kg)	3.24 (0.43)	3.25 (0.43)	0.2518
Length (cm)	50.5 (2.4)	50.9 (2.4)	0.0409
Age at visit (months)	26.1 (15.7)	19.8 (14.5)	0.0000
Weight (kg)	11.9 (3.6)	10.5 (3.3)	0.0000
Height (cm)	84.4 (14.6)	80.2 (14.3)	0.0000
W/H Z-scores	0.21 (1.3)	−0.04 (1.1)	0.0011
H/A Z-scores	−0.49 (1.4)	0.34 (0.73)	0.0000
W/A Z-scores	−0.12 (1.0)	0.02 (0.74)	0.3592
Intestinal parasites ^1^	2.81 (1.29)	2.90 (1.37)	0.4703
Hb (g/100 mL) ^2^	11.24 (0.8)	11.56 (0.8)	0.0000
Infant’s hair Hg (µg/g)	4.3 (1.7)	2.3 (1.2)	0.0000

W/H: Weight-for height; H/A: Height-for-age; W/A: Weight-for-age; Hb: Hemoglobin. ^1^ Testing positive for different species of intestinal parasites. ^2^ Due to sampling and/or analytical limitations these values represent different numbers of children, respectively (221) and (535). In order to compare and analyze statistically the villages, data were adapted from Marques et al. [17] and [18] respectively for Itapuã and Bom Futuro.
